# Parishin C Attenuates Oxidative Stress and Inflammation in HT22 Hippocampal Neurons and BV2 Microglia Through Nrf2 Signaling Pathway

**DOI:** 10.3390/ijms26157263

**Published:** 2025-07-27

**Authors:** Yichen Wang, Wenze Wu, Xinyan Wu, Basit Ali Shah, Mauro Lombardo, Gang Ye

**Affiliations:** 1 College of Life and Health Science, Northeastern University, Shenyang 110819, China; 2 College of Food Science and Nutritional Engineering, China Agricultural University, Beijing 100083, China; 3 School of Biomedical Engineering, Guangzhou Medical University, Guangzhou 511436, China; 4 Department for the Promotion of Human Science and Quality of Life, San Raffaele Open University, Via di Val Cannuta 247, 00166 Rome, Italy; 5 College of Veterinary Medicine, Sichuan Agricultural University, Chengdu 625014, China

**Keywords:** parishin C, bioactive compounds, antioxidant, anti–inflammatory, Nrf2, ferroptosis

## Abstract

Parishin C (PaC) is an active ingredient in *Gastrodia elata* Bl. that has neuroprotective effects. However, research on its role in oxidative stress and neuroinflammation is still limited. This study used LPS–stimulated HT22 cells to investigate the antioxidant properties of PaC. Through the co–culture system of HT22 and BV2 cells, the effect of PaC on neuroinflammation was explored. The current results indicated that PaC can inhibit the levels of reactive oxygen species and peroxides in LPS–stimulated HT22 cells and increase the levels of antioxidant factors. Meanwhile, PaC can also inhibit neuronal ferroptosis and the levels of pro–inflammatory cytokines in BV2 cells. Importantly, the antioxidant and anti–inflammatory effects of PaC are achieved by activating the Nrf2 signaling pathway. The WB and IF results indicated that PaC can promote nuclear translocation of Nrf2, activate downstream antioxidant factors, and thereby regulate inflammatory responses. Inhibition of Nrf2 can significantly inhibit the regulation of PaC on the Nrf2 signaling pathway. These results indicated that PaC can activate the Nrf2 signaling pathway to inhibit oxidative stress and inflammation.

## 1. Introduction

With the aggravation of social aging, neurodegenerative diseases have become one of the main reasons for the global morbidity rate and disability rate. Alzheimer’s disease (AD), Parkinson’s disease (PD), Huntington’s disease (HD), and other common neurodegenerative diseases have complex pathogenesis involving multiple molecular and cellular mechanisms. At present, the exact molecular mechanism regulating these diseases is still unclear. Many studies have shown that oxidative stress and neuroinflammation are widely involved in the occurrence and development of neurodegenerative diseases [1,2,3]. The occurrence of oxidative stress is due to the excess production of reactive oxygen species (ROS) exceeding the cellular antioxidant system, resulting in an imbalance between oxidation and antioxidants [4]. Oxidative stress can damage the proteins, lipids, and DNA, leading to cell death. Oxidative stress can also trigger inflammatory reactions, thereby accelerating cellular damage [5]. Ferroptosis is a type of cell death caused by the accumulation of large amounts of lipid ROS and iron. Glutathione peroxidase 4 (GPX4) is a key regulator of ferroptosis and the only substance in cells that can reduce phospholipid hydroperoxides [6]. GPX4 uses GSH as a substrate to reduce toxic lipid peroxides (PL–OOH) to non–toxic lipid alcohols (PL–OH) [7]. Regulating GPX4 to inhibit ferroptosis may be a beneficial measure for neurodegenerative diseases. Although iron is a substance that produces ATP in the brain, excessive iron and oxidative damage can have a significant impact on neural tissue. Therefore, regulating oxidative stress–dependent cell death and reducing corresponding inflammatory responses has therapeutic potential in neurodegenerative diseases.

Nuclear factor E2–related factor 2 (Nrf2) is a key transcription element involved in redox balance in the body [8]. Under normal conditions, Nrf2 exists in the cytoplasm and binds to Keap1 at low levels. When cells undergo oxidative stress, Keap1 undergoes conformational changes and releases Nrf2 to promote its translocation to the nucleus [9]. Nrf2 enters the nucleus and binds to ARE, promoting transcriptional activation and releasing downstream target genes. These target genes can participate in regulating iron and antioxidant systems to protect cells from ferroptosis caused by lipid peroxidation [10]. Nrf2 is a feasible therapeutic target for neurodegenerative diseases, which can participate in many pathological processes of neurodegenerative diseases, including oxidative stress and neuroinflammation [11]. Therefore, this study aims to explore the regulation of PaC on Nrf2 and its downstream genes.

Many natural products have been found to exhibit remarkable neuroprotective effects. *Gastrodia elata* Bl. (*G. elata*) is a precious traditional Chinese medicine with a long history recorded in the Pharmacopeia of Chinese Medicine. The earliest record of *G. elata* was in Shen Nong’s herbal classic, which was believed to have the effects of calming the wind, stopping spasms, and calming the liver yang [12]. *G. elata* has a wide range of pharmacological effects, including neuroprotective effects, anti–inflammatory effects, antioxidant effects, etc. [13]. More than 200 active ingredients have been isolated and identified from *G. elata*, including gastrodin, gastrodigenin, vanillin, parishin, and others. The phenolic compounds in *G. elata* are the basis for its pharmacological effects [14]. Parishin C (PaC) is the bioactive phenolic compound isolated and identified from *G. elata* [15]. In addition, PaC can exert antidepressant effects by inhibiting inflammatory responses. Importantly, PaC is one of the main components of *G. elata* against neuroinflammatory properties and has neuroprotective effects [16,17]. In the rat model of cerebral ischemia, PaC improved tissue damage after cerebral ischemia by regulating oxidative stress and inflammatory response [18]. Extensive research has mainly focused on gastrodin, and research on PaC is still not thorough. In particular, the efficacy and mechanism of PaC against neurodegenerative diseases are still unclear. In this study, LPS–stimulated HT22 and BV2 cells were used as models to investigate the effect of PAC on oxidative stress, neuroinflammation, and ferroptosis, providing more scientific evidence for the application of PaC.

## 2. Results

### 2.1. The Effect of PaC on LPS–Stimulated HT22 Cell Viability

**Figure 1:**
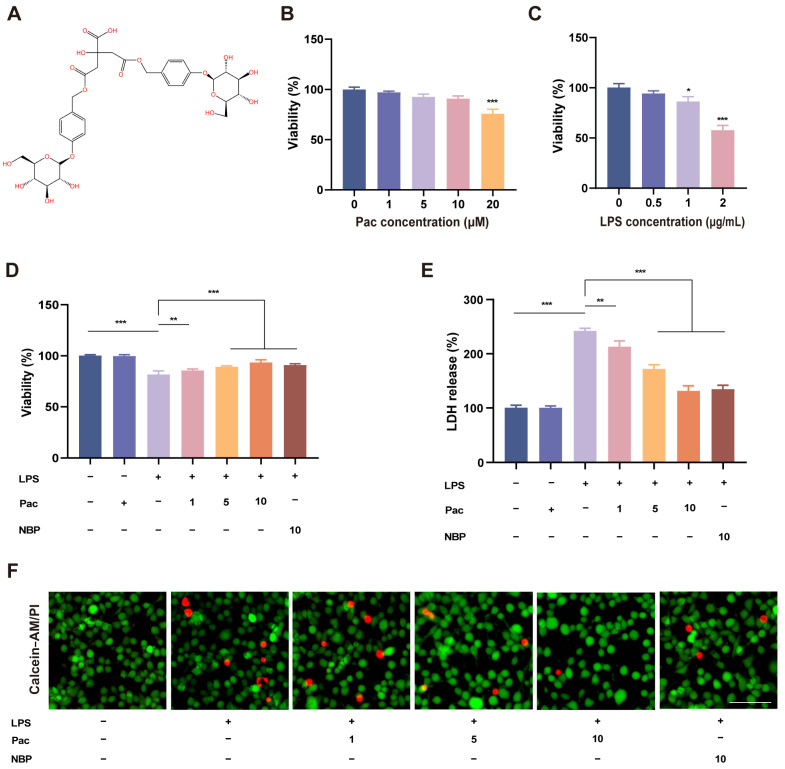
(**A**) The structure of PaC. (**B**) The effect of different concentrations of PaC on the viability of HT22 cells. *** *p* < 0.001, vs. the Ctrl group. (**C**) The effect of different concentrations of LPS on the viability of HT22 cells. * *p* < 0.05, *** *p* < 0.001, vs. the Ctrl group. (**D**) The effect of PaC on the viability of HT22 cells under LPS stimulation. (**E**) LDH release. (**F**) The Calcein AM– and PI–positive HT22 cells measured by fluorescence microscopy (bar = 50 μm). PaC: Parishin C; NBP: 3–N–Butylphthalide. Data are presented as the mean ± SD (n = 3). ** *p* < 0.01, *** *p* < 0.001.

PaC is a natural phenolic glucoside isolated from *G. elata*, and its chemical structure is shown in Figure 1A. We first investigated the effects of different concentrations of PaC on hippocampal neurons, HT22 cells. The MTT experiment results (Figure 1B) showed that when the PaC concentration was 20 μM, it could significantly reduce cell viability (*** *p* < 0.001). Therefore, the concentrations of PaC were 1, 5, and 10 μM for subsequent experiments. In order to select the appropriate concentration of LPS for cell modeling, we screened the stimulation concentrations of LPS (0.5, 1, 2 μg/mL). It was found that when the concentration of LPS was 2 μg/mL, the cell viability was only about 60% (Figure 1C). Therefore, we stimulated HT22 cells with 1 μg/mL LPS for 24 h as the treatment condition. Compared with Ctrl, LPS stimulation significantly reduced the cell viability and increased LDH levels of HT22 cells, while PaC treatment increased cell viability and inhibited LDH release, with concentration–dependent effects (Figure 1D,E). The NBP (3–N–Butylphthalide) can significantly increase cell viability and inhibit LDH release. NBP is a neuroprotective agent isolated from celery seeds that can activate Nrf2 to exert antioxidant and anti–inflammatory effects and was used as a positive control in this study [19]. The results of Calcein–AM/PI staining were consistent with the above results (Figure 1F). Therefore, PaC can reverse cell survival after LPS stimulation.

### 2.2. PaC Can Regulate Oxidative Stress

**Figure 2:**
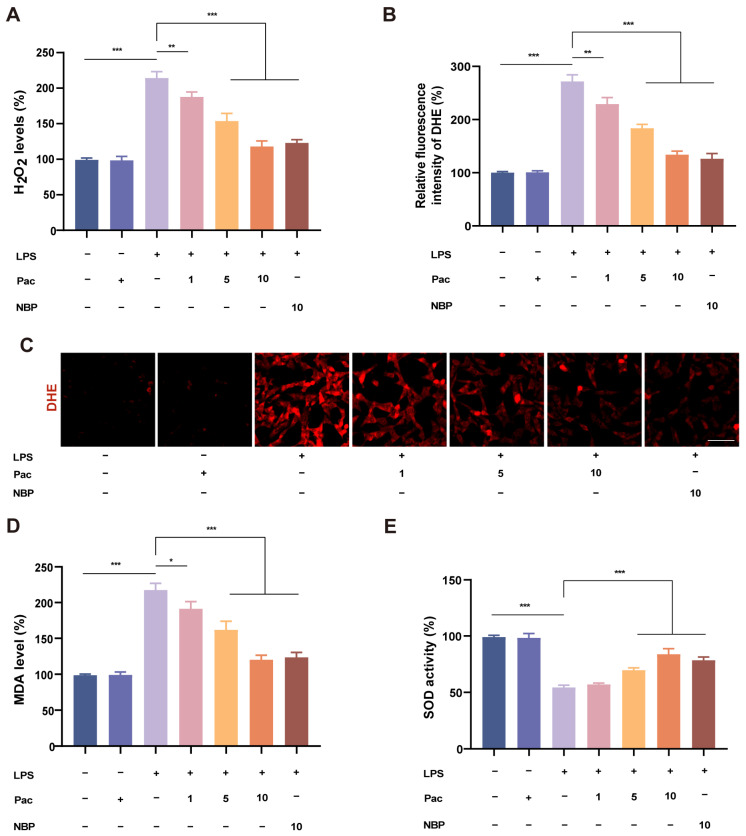
(**A**) The histogram of H_2_O_2_ content. (**B–C**) DHE fluorescence quantitative histogram and fluorescence diagram (bar = 20 μm). (**D**) MDA level. (**E**) SOD activity. PaC: Parishin C; NBP: 3–N–Butylphthalide. Data are presented as the mean ± SD (n = 3). * *p* < 0.05, ** *p* < 0.01, *** *p* < 0.001.

Oxidative stress is closely related to neurodegenerative diseases, and there are numerous studies on neuroprotective agents aimed at exploring their ability to resist neuronal ROS and prevent neurodegenerative diseases [20]. Therefore, this study mainly explored the neuroprotective mechanism of PaC in response to oxidative stress. We first explored the effect of PaC on ROS. The experimental results showed that PaC can resist the increase in H_2_O_2_ levels caused by LPS stimulation (Figure 2A). The DHE fluorescence staining results indicated that PaC can inhibit the level of superoxide anions (Figure 2B,C). PaC can also inhibit the level of MDA (Figure 2D). PaC can enhance cellular antioxidant capacity by increasing the levels of SOD (Figure 2E). The above results suggested that PaC had the ability to regulate oxidative stress.

### 2.3. PaC Can Activate the Nrf2 Signaling Pathway

**Figure 3:**
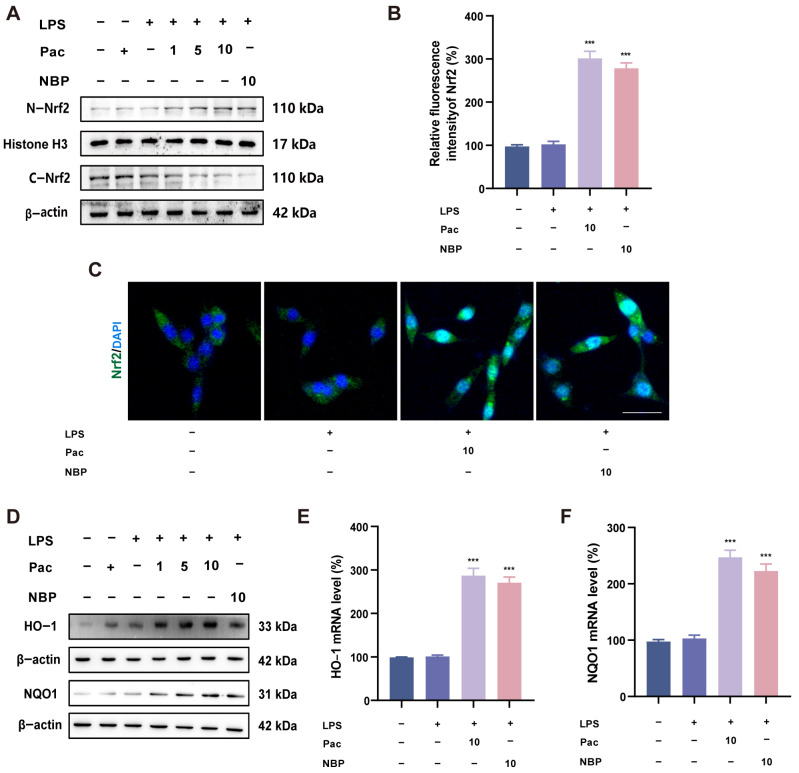
(**A**) WB results of N–Nrf2 and C–Nrf2. (**B**) Fluorescence quantification histogram of Nrf2. (**C**) Fluorescence diagram of Nrf2 in HT22 cells (green: Nrf2; blue: DAPI, bar = 10 μm). (**D**) WB results of HO–1 and NQO1. (**E**,**F**) The levels of HO–1 and NQO1 mRNA. PaC: Parishin C; NBP: 3–N–Butylphthalide. Data are presented as the mean ± SD (n = 3). *** *p* < 0.001, vs. the LPS group.

Nrf2 can participate in the expression of various antioxidant enzymes and is widely involved in regulating oxidative stress. Therefore, we explored whether PaC can activate the Nrf2 signaling pathway. The WB and IF results indicated that PaC can promote nuclear translocation of Nrf2 (Figure 3A–C). Meanwhile, PaC can activate the protein and mRNA levels of downstream antioxidant factors HO–1 and NQO1 (Figure 3D–F).

### 2.4. PaC Inhibited HT22 Cells Ferroptosis

**Figure 4:**
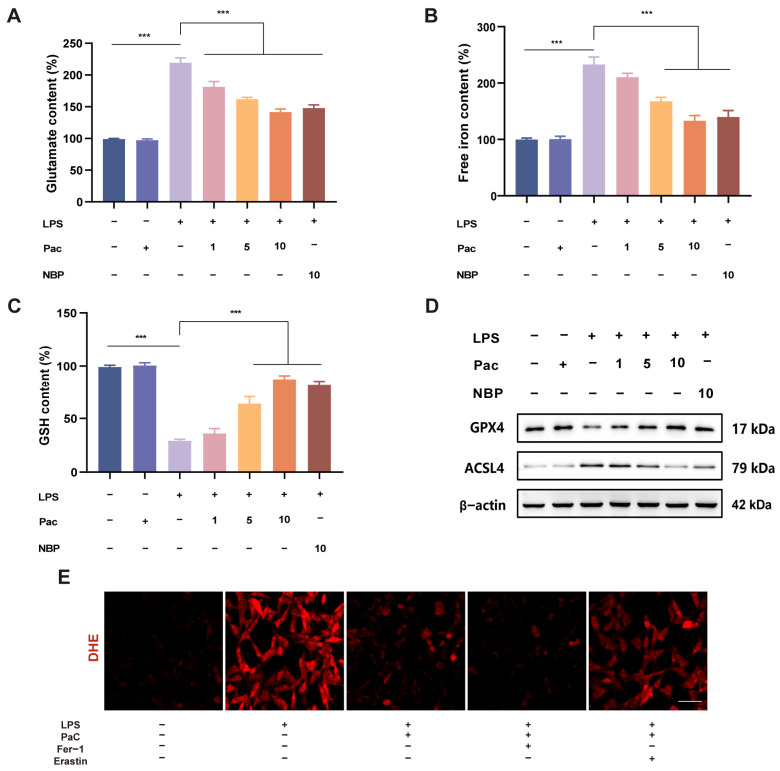
(**A**) Glutamate content. (**B**) Iron content. (**C**) GSH content. (**D**) WB results of ferroptosis related proteins GPX4 and ASCL4. (**E**) Fluorescence diagram of DHE after treatment with ferroptosis inhibitor Fer–1 and ferroptosis activator erastin (bar = 10 μm). PaC: Parishin C; NBP: 3–N–Butylphthalide. Data are presented as the mean ± SD (n = 3). *** *p* < 0.001.

**Figure 5:**
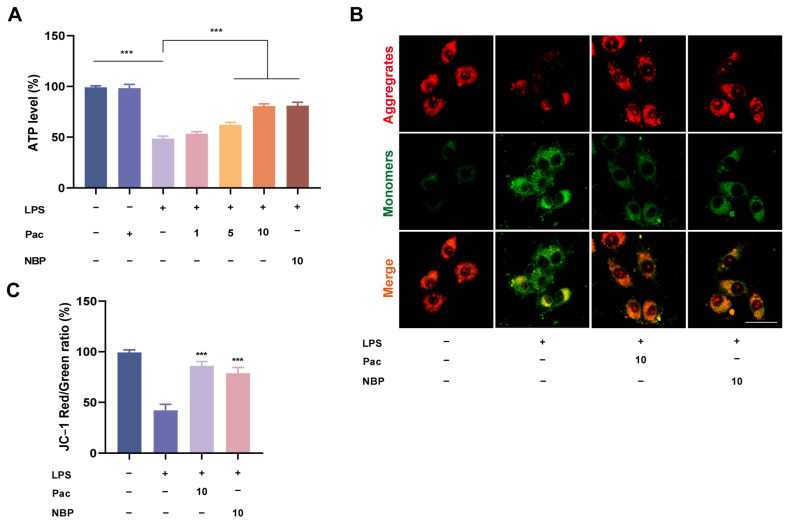
(**A**) ATP level. *** *p* < 0.001. (**B**) Mitochondrial membrane potential JC–1 fluorescence diagram (bar = 10 μm). (**C**) Histogram of JC–1 red/green ratio. PaC: Parishin C; NBP: 3–N–Butylphthalide. Data are presented as the mean ± SD (n = 3). *** *p* < 0.001, vs. the LPS group.

Oxidative stress is closely related to ferroptosis, and studies have found that Nrf2 can regulate lipid metabolism, iron, and energy metabolism, thereby participating in the regulation of ferroptosis [21]. Therefore, we further investigate whether PaC can participate in regulating neuronal ferroptosis. Mitochondria are the main site for producing ROS and are closely related to the occurrence of ferroptosis [22]. Therefore, we also tested the relevant indicators of mitochondria. The experimental results indicated that PaC can inhibit the increase in glutamate and free iron and increase GSH levels (Figure 4A–C). We detected the expression of ferroptosis–related proteins (GPX4 and ASCL4) and found that PaC can promote the expression of GPX4 while inhibiting the expression of ASCL4 (Figure 4D). To further verify the anti–ferroptosis effect of PaC, ferroptosis inhibitor Fer–1 and ferroptosis activator erastin were used to treat HT22 cells, and then superoxide anion levels were investigated. DHE fluorescence staining results (Figure 4E) showed that Fer–1 promoted the effect of PaC, while erastin reversed the effect of PaC. PaC improved the decrease in ATP levels caused by LPS (Figure 5A). The transition of JC–1 staining from red to green indicated a decrease in mitochondrial membrane potential, and PaC can improve this phenomenon (Figure 5B,C).

### 2.5. PaC Can Inhibit Neuroinflammation in BV2 Cells

**Figure 6:**
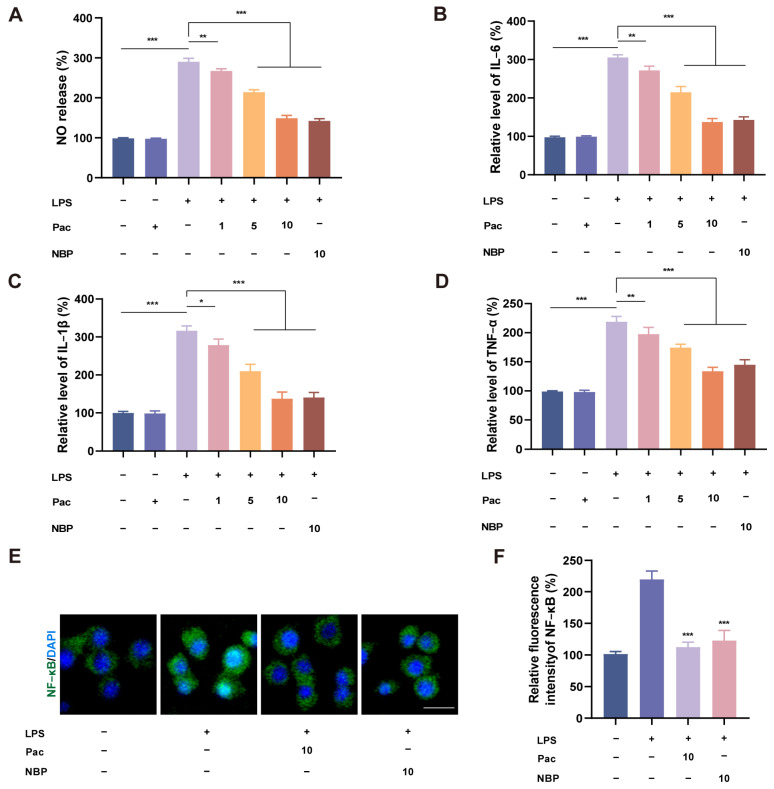
(**A**) The level of NO was detected in the LPS–stimulated BV2 cells. (**B**–**D**) PaC suppressed IL–6, IL–1β, and TNF–α mRNA levels. (**E**,**F**) Fluorescence diagram and fluorescence quantitative histogram of NF–κb in BV2 cells (green: NF–κb; blue: DAPI, bar = 10 μm). *** *p* < 0.001, vs. the LPS group. PaC: Parishin C; NBP: 3–N–Butylphthalide. Data are presented as the mean ± SD (n = 3). * *p* < 0.05, ** *p* < 0.01, *** *p* < 0.001.

Studies have shown that ferroptosis is also a pro–inflammatory mode of death, but the mechanism by which ferroptosis regulates inflammation is still unclear [23]. We co–cultured HT22 cells and BV2 cells using a transwell experiment to investigate the effect of PaC on the inflammatory response of microglia. Through Griess and qRT–PCR experiments (Figure 6A–D), we found that PaC can inhibit the level of NO and the mRNA levels of pro–inflammatory cytokines (IL–6, IL–1β, and TNF–α). The results of the IF indicated that PaC can reverse the nuclear translocation of the inflammatory regulatory factor NF–κb stimulated by LPS (Figure 6E,F). Based on these results, it is demonstrated that PaC can inhibit the inflammatory response of BV2 cells stimulated by LPS.

### 2.6. PaC Regulated Oxidative Stress and Inflammation by Targeting Nrf2

**Figure 7:**
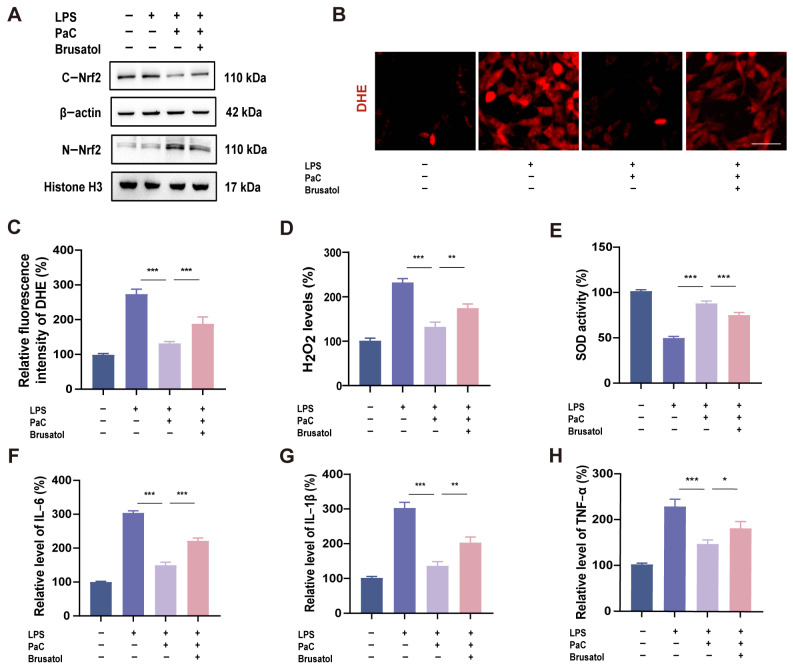
(**A**) WB results of C–Nrf2 and N–Nrf2. (**B**) DHE fluorescence diagram (bar = 10 μm). (**C**–**E**) The levels of DHE, H_2_O_2_, and SOD. (**F**–**H**) The mRNA levels of IL–6, IL–1β, and TNF–α. PaC: Parishin C. Data are presented as the mean ± SD (n = 3). * *p* < 0.05, ** *p* < 0.01, *** *p* < 0.001.

To investigate the role of Nrf2 in regulating oxidative stress and inflammatory response in PaC, we used Brusatol, a specific inhibitor of the Nrf2 pathway. After co-incubating cells with Brusatol, the nuclear translocation of Nrf2 was significantly reduced (Figure 7A). The inhibitory effects of PaC on DHE superoxide anion and H_2_O_2_ were also reversed by Brusatol (Figure 7B–D). Brusatol also inhibited the antioxidant activity of PaC, and after using Brusatol, SOD activity decreased (Figure 7E). The qRT–PCR results showed that the anti–inflammatory effect of PaC was weakened after inhibiting Nrf2 (Figure 7F–H). Therefore, Nrf2 was a key target for PaC to exert antioxidant and anti–inflammatory effects.

### 2.7. PaC Inhibited Ferroptosis by Targeting Nrf2

**Figure 8:**
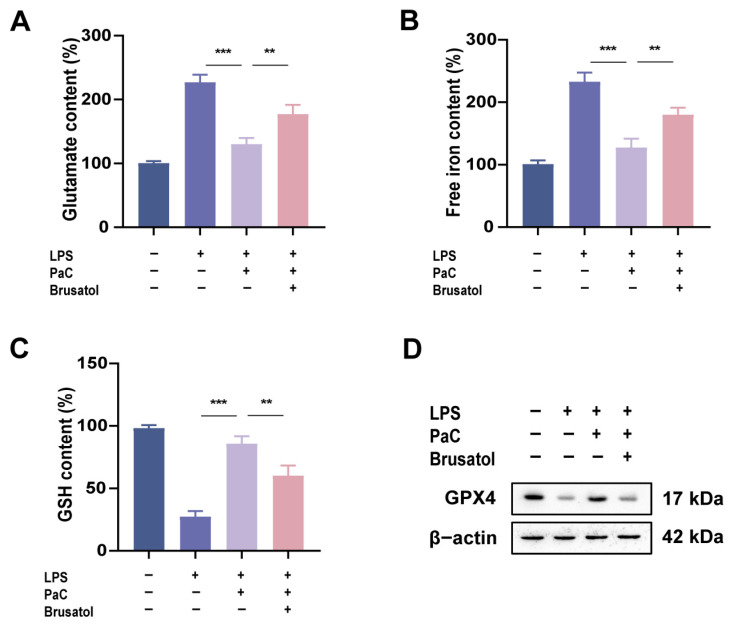
(**A**) Histogram of glutamate content. (**B**) Free iron content. (**C**) GSH content. (**D**) WB results of GPX4. PaC: Parishin C. Data are presented as the mean ± SD (n = 3). ** *p* < 0.01, *** *p* < 0.001.

The Nrf2 inhibitor Brusatol partially reversed the effects of PaC on glutamate, free iron, and GSH (Figure 8A–C). The role of PaC in promoting the expression of ferroptosis key protein GPX4 was also weakened by Brusatol (Figure 8D). These results indicated that PaC can regulate Nrf2 and inhibit neuronal ferroptosis.

### 2.8. Knocking Down Nrf2 Weakened the Neuroprotective Effect of PaC

**Figure 9:**
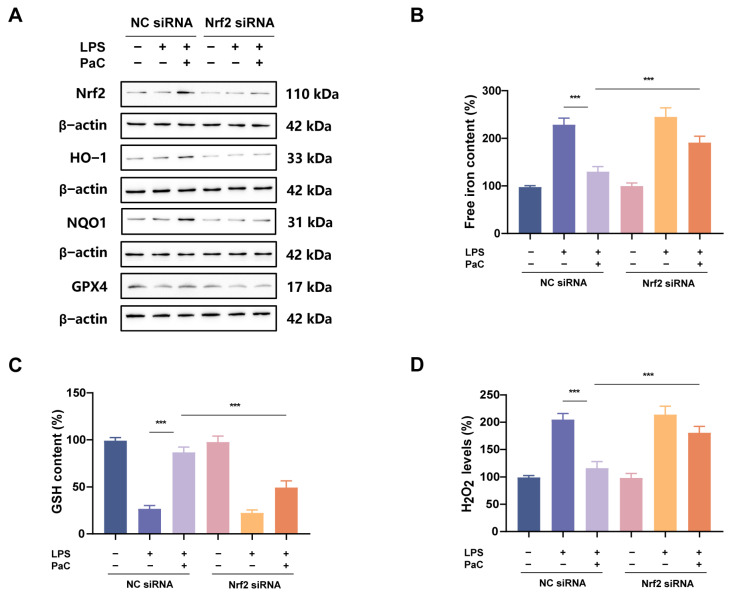
(**A**) WB analysis of Nrf2, HO–1, NQO1, and GPX4. (**B**) Free iron content. (**C**) GSH content. (**D**) H_2_O_2_ level. PaC: Parishin C. Data are presented as the mean ± SD (n = 3). *** *p* < 0.001.

Transfection of Nrf2 siRNA into HT22 cells was used to further validate the regulation of PaC on the Nrf2 signaling pathway. The experimental results showed that compared with the negative control group transfected with NC siRNA, cells transfected with Nrf2 siRNA significantly inhibited the expression of Nrf2, HO–1, NQO1, and GPX4 (Figure 9A). In addition, knocking down Nrf2 inhibited the effects of PaC on iron content, GSH levels, and H_2_O_2_ levels (Figure 9B–D).

## 3. Discussion

In this study, we demonstrated that PaC exhibits significant antioxidant and anti–inflammatory activities in LPS–stimulated HT22 cells and BV2 microglia. While previous studies have established the neuroprotective properties of PaC, its precise role in regulating oxidative stress and neuroinflammation has remained largely unexplored. Our findings provide new evidence that supports the regulatory role of PaC in redox homeostasis and neuroinflammation interactions.

Specifically, PaC significantly reduced the accumulation of ROS and lipid peroxides in HT22 cells, while concurrently upregulating key antioxidant factors such as HO–1 and NQO1, indicating its ability to mitigate oxidative stress and restore redox homeostasis. Moreover, PaC significantly suppressed ferroptosis, which is increasingly recognized as a contributor to neuronal damage under oxidative conditions. These findings suggest that PaC not only attenuates oxidative stress but also preserves neuronal viability by inhibiting ferroptotic pathways, thereby further underscoring its neuroprotective potential. Notably, recent studies have demonstrated that natural compounds targeting oxidative stress and ferroptosis can promote neuroprotection, thereby supporting our findings. For instance, Sun et al. [24] demonstrated that berberine alleviated AD by inhibiting the JNK–P38 MAPK signaling pathway, thereby enhancing autophagy and suppressing ferroptosis. This mechanism resulted in a significant reduction in amyloid–β plaque deposition, diminished neuroinflammation, and overall attenuation of neuronal damage. Similarly, Li et al. [25] reported that caffeic acid conferred neuroprotection in a rat model of permanent cerebral ischemia injury by inhibiting ferroptosis through activation of the Nrf2 antioxidant pathway, highlighting a key molecular axis in neuronal survival. Additionally, Guan et al. [26] provided a comprehensive review of the critical role of the antioxidant properties of polyphenols in neurodegenerative diseases. These studies collectively underscore the therapeutic potential of natural compounds, such as PaC, that target oxidative stress and ferroptosis to mitigate neuronal injury and promote brain health.

Furthermore, using a co–culture system of HT22 cells and BV2 microglia, we demonstrated that PaC significantly inhibited the production of pro–inflammatory cytokines in microglia, thereby highlighting its potential to alleviate neuroinflammatory responses. Mechanistically, these protective effects were closely associated with the activation of the Nrf2 signaling pathway. Western blot and immunofluorescence analyses further revealed that PaC promoted the nuclear translocation of Nrf2, which enhanced the expression of downstream antioxidant enzymes, including HO–1 and NQO1. Importantly, pharmacological inhibition of Nrf2 significantly reduced the antioxidant and anti–inflammatory effects of PaC, thereby further highlighting the crucial role of this pathway in mediating its neuroprotective effect. In fact, recent studies have increasingly demonstrated that Nrf2 serves as a crucial target through which natural compounds mediate their neuroprotective effects. For example, Li et al. [27] found that rutin effectively targeted Nrf2 to reverse neuronal damage, highlighting the therapeutic potential of Nrf2 activation in neurotoxicity. Similarly, our recent work revealed that inhibition or knockdown of Nrf2 abolished the antioxidant effects of 1,8–Cineole in corticosterone (CORT)–induced PC12 cells, thereby further confirming the indispensable role of Nrf2 in mediating the antioxidative efficacy of natural bioactive compounds [28]. Taken together, these results suggest that PaC’s ability to activate the Nrf2 signaling cascade constitutes a key mechanism underlying its dual antioxidative and anti–inflammatory neuroprotective effects. This finding supports the therapeutic potential of PaC, which targets Nrf2, in the prevention and treatment of neurodegenerative diseases characterized by oxidative stress and neuroinflammation.

Collectively, these findings suggest that PaC exerts neuroprotective effects by coordinately modulating oxidative stress, ferroptosis, and inflammation through activation of the Nrf2 signaling pathway. Given the increasing recognition of oxidative stress and neuroinflammation as central contributors to the pathogenesis of neurodegenerative diseases, PaC emerges as a promising therapeutic candidate, warranting further investigation in preclinical models. However, several limitations of the current study should be acknowledged. First, our experiments were primarily conducted in vitro using cell lines, which, while providing valuable mechanistic insights, may not fully recapitulate the complex cellular and molecular interactions present in the living brain. Therefore, comprehensive in vivo studies are necessary to validate the neuroprotective efficacy and safety profile of PaC in animal models of neurodegeneration. Second, although we demonstrated that PaC activates the Nrf2 pathway to exert antioxidative and anti–inflammatory effects, the upstream molecular targets and signaling cascades responsible for PaC–mediated Nrf2 activation remain to be elucidated. Elucidating these mechanisms could provide a deeper understanding of PaC’s pharmacodynamics and help identify additional therapeutic targets. Third, the potential effects of PaC on other regulated cell death pathways beyond ferroptosis, such as apoptosis or necroptosis, have not been explored. Further studies are warranted to comprehensively characterize its neuroprotective spectrum. Finally, issues related to pharmacokinetics, bioavailability, blood–brain barrier permeability, and potential off–target effects of PaC should be evaluated to assess its translational potential as a therapeutic agent. We are actively addressing these challenges to establish PaC’s therapeutic value and facilitate its clinical development for neurodegenerative diseases.

## 4. Materials and Methods

### 4.1. Chemicals, Reagents, and Antibodies

PaC was purchased from Macklin Biochemical Technology Co., Ltd. in Shanghai, China (purity 98%). 3–N–Butylphthalide (NBP, purity ≥ 98%) was obtained from Sigma–Aldrich (St. Louis, Mo, USA). MTT, LDH, Calcein–AM/PI assay kit, H_2_O_2_, MDA, SOD, GSH, DHE fluorescent probes, ATP assay kit, and JC–1 kit were obtained from Beyotime (Shanghai, China). Nrf2 (No. F111502), NQO1 (No. F104502), and β–actin (No. F115607) antibodies were provided by Abways Biotechnology Co., Ltd. (Shanghai, China). ASCL4 (No. T510198F) antibody was obtained from Abmart (Shanghai, China). HO–1 (No. 10701–1–AP) and NF–κb (No. 10745–1–AP) antibodies were purchased from Proteintech (Chicago, IL, USA). GPX4 (No. 399306) antibody was obtained from MedChemExpress (South Brunswick Township, NJ, USA).

### 4.2. Cell Culture

Mouse hippocampal neurons, HT22 cells, and mouse–derived microglia BV2 cells were purchased from a national experimental cell resource sharing platform. HT22 cells and BV2 cells were cultured in DMEM medium containing 10% fetal bovine serum (FBS), penicillin, and streptomycin. The cells were placed in a humidified incubator containing 5% CO_2_ at 37 °C.

### 4.3. Cell Survival Assessment

The construction of the HT22 cell model is stimulated with LPS when the cell density reaches 80%. Different concentrations of LPS (0.5, 1, and 2 ug/mL) were applied to cells to investigate the optimal concentration of LPS. After treating cells with LPS for 24 h, cell viability was detected using the MTT assay. The specific method of MTT was based on existing research [29].

After applying the most suitable LPS stimulation conditions to HT22 cells, different concentrations of PaC (1, 5, and 10 μM) and 3–N–Butylphthalide (NBP, 10 μM) were added. Subsequently, MTT and LDH were used to detect cell survival. The Calcein–AM/PI cell viability and cytotoxicity assay kit was also used to evaluate the effect of PaC on cell survival. The detection method was described in the kit manual.

### 4.4. Oxidative Stress Assessment

We investigated indicators of oxidative stress, including H_2_O_2_, MDA, SOD, and GSH. We conducted the specific experimental operation according to the instructions of the reagent kits. In addition, we also used DHE fluorescent probes to detect superoxide anion levels. DHE fluorescent probe (10 μM) was added to the cells and incubated at 37 °C for 30 min. After staining, the cells were washed with PBS and observed under a fluorescence microscope.

### 4.5. Ferroptosis Assessment

The commercial test kits were used to detect indicators related to ferroptosis, including an enhanced ATP assay kit, the glutamate detection kit, and the iron assay kit. We conducted specific experiments according to the instructions of the reagent kits.

### 4.6. Mitochondrial Membrane Potential

The JC–1 kit was used to detect mitochondrial membrane potential. JC–1 staining solution was prepared according to the instructions of the kit and added to the cells. The cells were incubated at 37 °C for 30 min. After staining, the cells were washed with buffer solution and observed under a fluorescence microscope. The color change in JC–1 from red to green indicated a decrease in mitochondrial membrane potential.

### 4.7. Western Blot

Protein samples were extracted from cells using lysis buffer. After separating the protein sample by SDS–PAGE, it was transferred onto a PVDF membrane. 5% skim milk was sealed for 1 h, and then the PVDF membrane was incubated overnight with primary antibody at 4 °C. The primary antibodies we used include Nrf2, HO–1, NQO1, GPX4, ASCL4, and β–actin. After the incubation of the primary antibodies was completed, TBST was washed three times, and then the secondary antibodies were added. The chemiluminescence method was used for imaging protein bands.

### 4.8. Quantitative RT–PCR

After extracting RNA from each group, the RNA was reverse transcribed into DNA using a kit (Invitrogen, Carlsbad, California, USA). The primer sequences are shown in Table 1. The PCR process was used for quantitative detection of gene expression, followed by 40 cycles at 95 °C for 15 s, 60 °C for 30 s, and 72 °C for 30 s.

### 4.9. Immunofluorescence (IF)

HT22 and BV2 cells were fixed with paraformaldehyde and blocked with 5% goat serum for 1 h. Nrf2 and NF–κb antibodies were added to the cells and incubated overnight. After washing the cells with PBS, the 488–labeled goat anti–rabbit IgG (H + L) secondary antibody was incubated in the dark for 1 h, and then DAPI was added and observed under a fluorescence microscope.

### 4.10. HT22 and BV2 Co–Culture System

To observe the effect of HT22 cells on BV2 cells after LPS stimulation, we seeded HT22 cells in the upper layer of the transwell culture chamber and cultured BV2 cells at the bottom. In the same transwell chamber, the semi–permeable membrane allows for the sharing of culture medium and components between the upper and lower chambers.

### 4.11. NO Release

The assessment of NO levels referred to previous studies [30]. Specifically, under the stimulation of LPS, cells were treated with different concentrations of PaC. The cell supernatant was extracted, and an equal volume of Griess reagent was added under dark conditions. After incubation at 37 °C for 10 min, OD_540_ was measured using a microplate reader.

### 4.12. Knock Down Nrf2

Nrf2 siRNA (sense, GAAUGGUCCUAAAACACCAtt; antisense, UGGUGUUUUAGGACCAUUCtg) was designed and synthesized from GenePharma (Shanghai, China). LipoRNAi^TM^ (Beyotime, Shanghai, China) was used to transfect Nrf2 siRNA into HT22 cells for 48 h.

### 4.13. Statistical Analysis

Data are expressed as mean ± SD. All statistical analysis was performed with SPSS 22.0 software. Statistical analysis was performed by using one–way analysis of variance (ANOVA), and homogeneity of variance was tested, followed by Tukey’s or Dunnett’s T3 test. *p* < 0.05 was considered statistically significant.

## 5. Conclusions

In summary, this research found that PaC can improve cell damage under LPS stimulation by inhibiting oxidative stress and inflammation. Meanwhile, PaC also has an inhibitory effect on ferroptosis. These provided the basis for the application of PaC in neurodegenerative diseases.

## Figures and Tables

**Table 1:** The sequences of primers used for qRT–PCR.

Gene	Primer Sequence
HO–1	F: 5′–ACCATATCTACACGGCCCTG–3′ R: 5′–CAGTGAGGCCCATACCAGAA–3′
IL–6	F: 5′–TAGTCCTTCCTACCCCAATTTCC–3′ R: 5′–TTGGTCCTTAGCCACTCCTTC–3′
IL–1β	F: 5′–TGACGGACCCCAAAAGATGA–3′ R: 5′–TCTCCACAGCCACAATGAGT–3′
TNF–α	F: 5′–CCCTCACACTCAGATCATCTTCT–3′ R: 5′–GCTACGACGTGGGCTACAG–3′
GAPDH	F: 5′–AGGTCGGTGTGAACGGATTTG–3′ R: 5′–TGTAGACCATGTAGTTGAGGTCA–3′

## Data Availability

Data are contained within the article.
